# Aberrant activation of the mTOR pathway and anti-tumour effect of everolimus on oesophageal squamous cell carcinoma

**DOI:** 10.1038/bjc.2012.36

**Published:** 2012-02-14

**Authors:** K Hirashima, Y Baba, M Watanabe, R-I Karashima, N Sato, Y Imamura, Y Nagai, N Hayashi, K-I Iyama, H Baba

**Affiliations:** 1 Department of Gastroenterological Surgery, Graduate School of Medical Sciences, Kumamoto University, 1-1-1 Honjo, Kumamoto 860-8556, Japan; 2 Department of Surgical Pathology, Kumamoto University Hospital, 1-1-1 Honjo, Kumamoto 860-8556, Japan

**Keywords:** everolimus, RAD001, mTOR, oesophageal squamous cell carcinoma, proliferation

## Abstract

**Background:**

The mammalian target of rapamycin (mTOR) protein is important for cellular growth and homeostasis. The presence and prognostic significance of inappropriate mTOR activation have been reported for several cancers. Mammalian target of rapamycin inhibitors, such as everolimus (RAD001), are in development and show promise as anti-cancer drugs; however, the therapeutic effect of everolimus on oesophageal squamous cell carcinoma (OSCC) remains unknown.

**Methods:**

Phosphorylation of mTOR (p-mTOR) was evaluated in 167 resected OSCC tumours and 5 OSCC cell lines. The effects of everolimus on the OSCC cell lines TE4 and TE11 *in vitro* and alone or in combination with cisplatin on tumour growth *in vivo* were evaluated.

**Results:**

Mammalian target of rapamycin phosphorylation was detected in 116 tumours (69.5%) and all the 5 OSCC cell lines. Everolimus suppressed p-mTOR downstream pathways, inhibited proliferation and invasion, and induced apoptosis in both TE4 and TE11 cells. In a mouse xenograft model established with TE4 and TE11 cells, everolimus alone or in combination with cisplatin inhibited tumour growth.

**Conclusion:**

The mTOR pathway was aberrantly activated in most OSCC tumours. Everolimus had a therapeutic effect both as a single agent and in combination with cisplatin. Everolimus could be a useful anti-cancer drug for patients with OSCC.

Oesophageal squamous cell carcinoma (OSCC), the major histological type of oesophageal cancer in East Asian countries, is one of the most aggressive malignant tumours (Enzinger and Mayer, 2003). Despite the development of multimodal therapies, including surgery, chemotherapy, and radiotherapy, the prognosis remains poor even for patients who undergo complete carcinoma resection. The limited improvement in outcomes achieved by conventional therapies urges us to seek innovative strategies, especially those involving molecular targeting, for treating OSCC.

The mammalian target of rapamycin (mTOR) is a 289-kDa serine/threonine kinase involved in cellular growth and homeostasis (Bjornsti and Houghton, 2004; Abraham and Gibbons, 2007; Menon and Manning, 2008; Wouters and Koritzinsky, 2008). Mammalian target of rapamycin is activated by phosphorylation as a part of the phosphatidylinositol-3 kinase/AKT signalling pathway (Mita *et al*, 2003; Chan, 2004; Dancey, 2006) and in turn phosphorylates and activates eukaryotic translation factor 4E (elF4E) and p70 ribosomal S6 kinase (p70S6 kinase), leading to the translation of proteins required for cell cycle progression (Hidalgo and Rowinsky, 2000; Panwalkar *et al*, 2004). The presence and prognostic significance of aberrant mTOR activation have been reported for several types of human carcinomas (Easton and Houghton, 2006; Herberger *et al*, 2007; Hou *et al*, 2007; Hudes, 2009; Hirashima *et al*, 2010). Our group previously showed an association between high phosphorylated mTOR (p-mTOR) expression and poor prognosis in 143 resected OSCC samples (Hirashima *et al*, 2010). Mammalian target of rapamycin has recently been recognised as an important and attractive target for anti-cancer therapy (Boulay *et al*, 2004; Bianco *et al*, 2006; Johnston, 2006; Antonarakis *et al*, 2010; Sparks and Guertin, 2010). Everolimus, an oral mTOR inhibitor, has shown particularly promising results in experimental studies, inhibiting tumour growth and displaying anti-angiogenic effects (Carmeliet and Jain, 2000; Bianco *et al*, 2008; Manegold *et al*, 2008; Lane *et al*, 2009). Combination therapy using everolimus and cisplatin has also been reported to be effective (Beuvink *et al*, 2005; Mabuchi *et al*, 2007; Hou *et al*, 2010; Ma *et al*, 2010). Many clinical trials using everolimus for several types of cancers are currently underway (Yee *et al*, 2006; Fouladi *et al*, 2007; Gridelli *et al*, 2007; Johnson *et al*, 2007; Awada *et al*, 2008; O’Donnell *et al*, 2008; Tabernero *et al*, 2008; Tanaka *et al*, 2008; Yao *et al*, 2008; Wolpin *et al*, 2009). Everolimus has been already approved for the treatment of advanced renal cell carcinoma in patients whose disease has progressed during or after VEGF-targeted therapy (Coppin, 2010). Nonetheless, to our knowledge, no study has examined the therapeutic effect of everolimus on OSCC using *in vitro* and *in vivo* assays.

We therefore conducted this study with three main aims. First, we examined the importance of mTOR activation in OSCC by determining the overall prevalence of p-mTOR expression in OSCC specimens and cell lines. Second, we evaluated the therapeutic effect of everolimus on OSCC cell lines by both *in vitro* and *in vivo* assays. Third, we specifically assessed the effect of everolimus in combination with cisplatin, which is one of the most frequently used chemotherapeutic drugs, on OSCC cells.

## Materials and methods

### Reagents and antibodies

Everolimus was provided by Novartis Pharma AG (Basel, Switzerland) and formulated at 2% (w/v) in a microemulsion vehicle. For *in vivo* analysis, everolimus was diluted to the appropriate concentration in double-distilled water just before administration by gavage. For *in vitro* analyses, everolimus was prepared in DMSO just before addition to cell cultures. Antibodies recognising mTOR, phospho-mTOR (Ser^2448^), p70s6k, phospho-p70s6k (Thr^389^), 4E-BP1, phospho-4E-BP1 (Thr^70^), and *β*-actin were purchased from Cell Signaling Technology (Boston, MA, USA).

### Patients

The present study involved 167 consecutive patients who underwent surgical resection of OSCC at the Kumamoto University Hospital from January 1996 to December 2007. None of these patients underwent endoscopic mucosal resection, palliative resection, preoperative chemotherapy, preoperative radiotherapy, or preoperative chemoradiotherapy. This study was approved by the Institute Review Board of the Graduate School of Medical Science, Kumamoto University (Approval number: 236; 2 August 2008).

### Immunohistochemistry for p-mTOR

The method of immunohistochemical staining for p-mTOR was described previously (Hirashima *et al*, 2010). Of the 167 tumours, 51 showed no p-mTOR expression, 84 showed weak expression, and 32 showed strong expression. As the aim of the immunohistochemistry in this study was to evaluate the prevalence of p-mTOR expression in OSCC tissues, both weak and strong p-mTOR expression were defined as positive.

### Cell culture

Oesophageal squamous cell carcinoma cell lines (TE series) were obtained from the Cell Resource Center for Biomedical Research, Tohoku University. Cell cultures were grown in the recommended medium with 10% foetal bovine serum and incubated in 5% CO_2_ at 37 °C.

### Western blot analysis

Cultured cells were harvested and lysed in lysis buffer (25 mM Tris-HCl (pH 7.4), 100 mM NaCl, 2 mM EDTA, 1% Triton X-100, leupeptin, 1 mM Na_3_VO_4_, and 1 mM PMSF) for 30 min. Lysates were centrifuged at 10 000 rpm for 5 min at 4 °C. Each protein sample (10 *μ*g) was mixed with 5 × sample buffer containing 10% *β*-mercaptoethanol and boiled for 5 min. The total cellular protein extracts were separated by sodium dodecyl sulphate polyacrylamide gel electrophoresis (SDS–PAGE) on 7.5% gels for the examination of mTOR and p-mTOR, and on 12.5% gels for the examination of p70S6K, p-p70S6K, 4E-BP1, p-4E-BP1, and *β*-actin. The samples were then transferred to PVDF membranes (Bio-Rad, Hercules, CA, USA), which were blocked overnight at 4 °C in 5% skim milk in phosphate-buffered saline (PBS) containing 0.1% Tween 20. The membranes were probed overnight at 4 °C with each primary monoclonal antibody followed by incubation with peroxidase-conjugated anti-rat IgG antibody (1 : 1000) (Sigma, St Louis, MO, USA). The targets were detected using an enhanced chemiluminescence (ECL) reagent (GE Healthcare, Piscataway, NJ, USA).

### Cell proliferation analysis

The effect of everolimus on cell proliferation was evaluated using a water-soluble tetrazolium salt (WST-8; (2-(2-methoxy-4-nitrophenyl)-3-(4-nitrophenyl)-5-(2, 4-disulfophenyl)-2H-tetrazolium, monosodium salt) (Dojin Chemicals, Tokyo, Japan). TE4 and TE11 cells were cultured overnight in 96-well plates (3 × 10^3^ cells per well). Cells were then treated for 48 h with everolimus (20 nM) or vehicle (control) and their viabilities were assessed. The number of surviving cells in each sample was determined from its absorbance at 450 nm (A_450_).

### Cell cycle analysis

The cell cycle distribution of TE4 and TE11 cells treated with everolimus (20 nM) or vehicle (control) for 48 h was analysed by flow cytometry using a BD FACSCalibur (BD Bioscience, San Jose, CA, USA) according to previously published methods (Del Bufalo *et al*, 2004; Milella *et al*, 2004).

### Apoptosis analysis

TE4 and TE11 cells were treated with everolimus (20 nM) or vehicle (control) for 48 h and then apoptosis was assessed by flow cytometry using Annexin V-FITC (BD Bioscience) and propidium iodide (PI) staining according to previously published methods (Del Bufalo *et al*, 2004; Milella *et al*, 2004).

### Invasion analysis

To evaluate the effect of everolimus on cell invasiveness, a Matrigel Invasion Chamber (BD Bioscience) was used according to the manufacturer's protocol. Matrigel-coated chambers containing 8 *μ*m pore-size filters were fitted into 24-well tissue culture plates. Briefly, cells of each type (TE4, 1.0 × 10^5^ cells ml^−1^; TE11, 5.0 × 10^5^ cells ml^−1^) were seeded into the Matrigel-coated chambers in RPMI-1640 medium with everolimus (20 nM) or vehicle (control) and incubated at 37 °C in 5% CO_2_ for 24 h. The invasive cells on the bottom sides of the filters were stained using Toruijin blue dye, and the numbers of cells in five randomly selected fields at × 200 magnification were counted.

### Subcutaneous xenograft model

All the procedures involving animals and their care were approved by the Animal Care and Use Committee of Kumamoto University. These procedures meet the standards required by the United Kingdom Coordinating Committee for Cancer Research (UKCCCR) guidelines (Workman *et al*, 2010). A subcutaneous xenograft model was used to assess the therapeutic effect on OSCC cells of everolimus either as a single agent or in combination with cisplatin, one of the chemotherapeutic drugs most frequently used for OSCC in the clinical setting. Six-week-old nude mice (BALB/c) (*n*=24) were inoculated subcutaneously in the right or left flank with 5 × 10^6^ TE4 cells and TE11 cells in 200 *μ*l of PBS. Some mice showed insufficient tumour growth and were therefore excluded from the study, leaving a total of 22 mice used for the single *in vivo* experiment. When the tumours reached approximately 50–70 mm^3^, the mice were randomised into four treatment groups (*n*=5–6 mice per group). The first group was treated twice a week with placebo. The second group was treated twice a week with everolimus (5 mg kg^−1^). The third group was treated every 2 weeks with cisplatin (3 mg kg^−1^). The fourth group was treated twice a week with everolimus (5 ml kg^−1^) and every 2 weeks with cisplatin (3 mg kg^−1^) (Figure 4A). The validity of these everolimus and/or cisplatin protocols has been demonstrated in an ovarian cancer model. Everolimus was administered by oral gavage using an animal-feeding needle. Cisplatin was injected intraperitoneally. Body weight was measured every 3 days. Calliper measurements of the longest perpendicular tumour diameters were made weekly using a digital calliper, and tumour volumes were estimated using the following formula: *V*=*L* × *W* × *D* × *π*/6, where *V* is the tumour volume, *L* the length, *W* the width, and *D* the depth (Mabuchi *et al*, 2007).

### Statistical analysis

For the *in vitro* assays, including the cell proliferation assay, cell cycle ratio assay, apoptosis assay, and invasion assay, statistical analyses were performed using Mann–Whitney's *U*-test for unpaired samples. For the *in vivo* experiment, body weight and tumour volume were compared among placebo-, everolimus-, cisplatin-, and everolimus plus cisplatin-treated mice using the Wilcoxon exact test. Statistical analysis was performed with Stat View-J 5.0 software (Abacus Concepts, Inc., Berkeley, CA, USA). A two-sided significance level of *P*<0.05 was used for all the statistical analyses.

## Results

### Phosphorylated mTOR expression in OSCC specimens and cell lines

**Figure 1:**
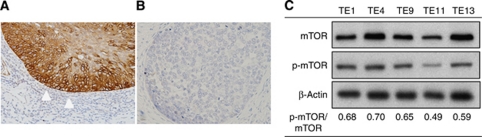
Immunostaining for p-mTOR. (**A**) Oesophageal squamous cell cancer cells positive for p-mTOR (white arrow). (**B**) Oesophageal squamous cell cancer cells negative for p-mTOR. (**C**) Western blot analysis of mTOR, p-mTOR, and *β*-actin levels in TE1, 4, 9, 11, and 13 cell lines.

We assessed p-mTOR expression (i.e., mTOR activation) by immunohistochemistry. Of the 167 OSCC specimens, 116 (70%) were positive for p-mTOR expression (Figures 1A and B). The high percentage of p-mTOR-positive tumours supports the crucial role of mTOR activation in the pathogenesis of OSCC.

All five human OSCC cell lines (TE1, 4, 9, 11, and 13) examined in the current study showed p-mTOR expression *in vitro*; the expression level was highest in TE4 cells and lowest in TE11 cells (Figure 1C). Therefore, both TE4 and TE11 cells were used in the following experiments.

### Everolimus attenuates phosphorylation of p70S6K and 4E-BP1 *in vitro*

**Figure 2:**
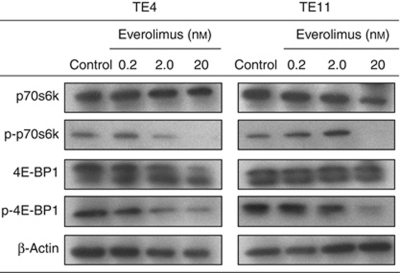
Western blot analysis for p70S6k, p-p70S6k, 4E-BP1 p-4E-BP1, and *β*-actin protein levels in TE4 and TE11 cells treated with (at indicated concentrations) or without everolimus.

The TE4 and TE11 cells were treated with different concentrations of everolimus (0 (vehicle control), 0.2, 2, and 20 nM) and the levels and phosphorylation of downstream mTOR targets, including p70S6k, p-p70S6k, 4E-BP1, p-4E-BP1, and *β*-actin (loading control), were evaluated by western blotting. Everolimus inhibited phosphorylation of p70S6k and 4E-BP1 (decreased levels of p-p70S6k and p-4E-BP1) in TE4 cells in a dose-dependent manner (Figure 2). In TE11 cells, 20 nM everolimus was sufficient to block phosphorylation of p70S6k and 4E-BP1 (Figure 2). Therefore, TE4 and TE11 cell lines were treated with 20 nM everolimus in the following assays (e.g., the *in vitro* proliferation, cell cycle, apoptosis, and invasion assays).

### Therapeutic effect of everolimus on OSCC cell lines *in vitro*

**Figure 3:**
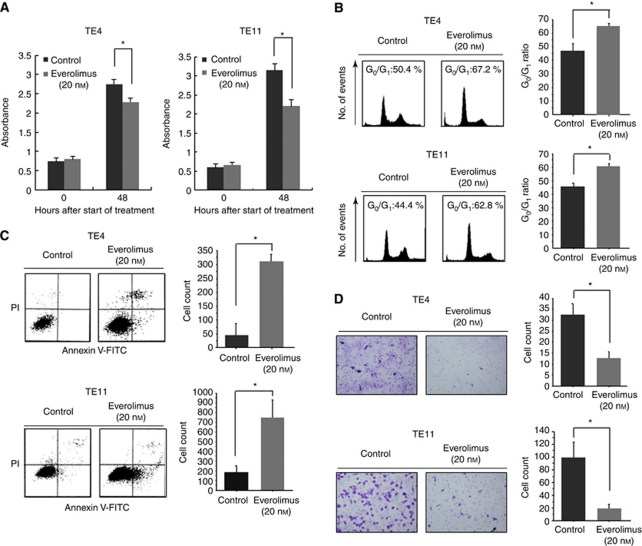
*In vitro* assay for confirming the anti-cancer activity of everolimus. (**A**) *In vitro* proliferation assay. Treatment with everolimus (20 nM) for 48 h decreased the proliferation ratios of both TE4 and TE11 cells compared with those of control vehicle-treated cells. ^*^P<0.05. (**B**) *In vitro* cell cycle assay. Treatment with everolimus (20 nM) increased the percentages of TE4 and TE11 cells in G_0_/G_1_ phase compared with those of control vehicle-treated cells. ^*^P<0.05. (**C**) *In vitro* cell apoptosis analysis. Induction of early apoptosis in TE4 and TE11 cells by everolimus is shown (lower right part; Annexin V-FITC-positive, PI-negative). (**D**) *In vitro* invasion assay. Everolimus (20 nM) decreased the numbers of invading TE4 and TE11 cells compared with those of control vehicle-treated cells ( × 200 magnification, five fields). ^*^P<0.05.

Everolimus (20 nM) treatment for 48 h significantly inhibited the proliferation of both TE4 and TE11 cells (Figure 3A). In order to clarify the effect of everolimus on the cell cycle, OSCC cells were treated with everolimus (20 nM) and then subjected to cell cycle analysis by flow cytometry. An accumulation of cells in the G_0_/G_1_ phase and a reduction in the S-phase fraction were observed in both TE4 and TE11 cells treated with everolimus (20 nM) for 48 h (Figure 3B). Everolimus (20 nM) also significantly increased the proportion of early apoptotic cells (Annexin V-FITC positive, PI negative) compared with that of vehicle-treated cells in both TE4 and TE11 cells treated for 48 h (Figure 3C), indicating that everolimus could induce early apoptosis in these cell lines. Western blot analysis utilising antibodies for Bad and PARP also showed the induction of apoptosis by everolimus (Supplementary Figure 1); everolimus (20 nM) increased the expression of Bad and cleaved PARP protein. Finally, we performed an *in vitro* invasion assay using Matrigel Invasion Chambers and found that everolimus (20 nM) significantly decreased the numbers of invading TE4 and TE11 cells compared with those of vehicle-treated cells (Figure 3D).

### Everolimus inhibits tumour growth in a mouse subcutaneous xenograft model

**Figure 4:**
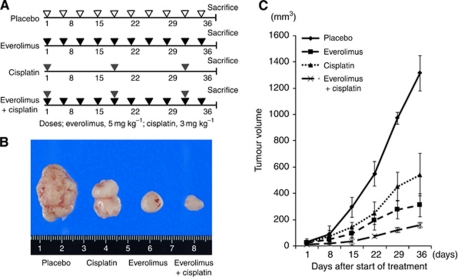
*In vivo* assay for confirming the anti-cancer activity of everolimus utilising a mouse xenograft model established with TE4 cells. (**A**) Treatment schedules for the four treatment groups (placebo, everolimus, cisplatin, and everolimus plus cisplatin). (**B**) Tumour volume in the four treatment groups (placebo, everolimus, cisplatin, and everolimus plus cisplatin) after the 5-week course of treatment. (**C**) Growth of tumour volume in the four treatment groups.

The mean tumour volumes on day 36 in a mouse xenograft model established with TE4 cells were 1314±134, 311±87, 542±161, and 159±21 mm^3^ in mice treated with placebo, everolimus, cisplatin, and everolimus plus cisplatin, respectively (Table 1, Figure 4B). Treatment with everolimus or cisplatin alone decreased the tumour burdens by 83% and 68%, respectively, compared with that of placebo-treated mice (Figure 4C), indicating that everolimus used as a single agent has marked anti-tumour activity. Moreover, treatment with cisplatin plus everolimus decreased the tumour burden by 92% (Figure 4C), suggesting that the use of everolimus and cisplatin as a combination therapy might be promising. Similar results were obtained for TE11 cells (Supplementary Figure 2).

The weight changes of the mice over the course of the treatments did not differ significantly among the four groups, as shown in Supplementary Figure 3. In addition, we confirmed histologically that there were no differences in the levels of injury to the organs, including liver, kidney, pancreas, lung, intestine, and skin, among these four groups (Supplementary Figure 4). We continued to follow these mice for 2 months. Although all of the mice in the placebo group died within 2 months, no mouse in any of the other three groups died during this period.

## Discussion

Mammalian target of rapamycin is a key regulator of cell growth and proliferation and as such is regarded as a promising target for anti-cancer therapy (Kapoor, 2009; Scott *et al*, 2009). In this study, we made three intriguing findings. First, most OSCC tumours were positive for p-mTOR expression, supporting a role for mTOR activation in the pathogenesis of OSCC. Second, everolimus, an oral mTOR inhibitor, had a therapeutic effect on OSCC cell lines *in vitro*. Third, combination therapy with everolimus and cisplatin showed an additive effect on OSCC cells *in vivo*. Our findings certainly suggest that everolimus could be useful as an anti-cancer drug for patients with OSCC.

Previous studies have shown the importance of mTOR activation in OSCC specimens: Boone *et al*, (2008) detected activated mTOR in 25% of patients with OSCC, a subset of patients that might potentially benefit from mTOR-inhibiting therapy. Yoshioka *et al*, (2008) demonstrated that 48% of OSCC tumours showed high levels of p-mTOR phosphorylation. In the current study utilising 167 OSCC samples, about 70% of the OSCC tumours showed p-mTOR (i.e., mTOR activation). This discrepancy might be due to a difference in the method used to evaluate mTOR phosphorylation or in the cutoff for p-mTOR positivity. Nonetheless, these two previous studies and the current study certainly support the hypotheses that mTOR activation is important in the pathogenesis of OSCC and that mTOR inhibitors might be useful for OSCC treatment.

Everolimus, an orally bioavailable derivative of rapamycin, is a promising drug for cancer therapy. However, to our knowledge, no previous study has utilised *in vitro* and *in vivo* models to evaluate the therapeutic efficacy of everolimus. First, we demonstrated that everolimus suppressed down-stream signalling (i.e., phosphorylation of p70s6 kinase and 4E-BP1) and significantly inhibited cell proliferation and invasion of mTOR-activated OSCC cell lines *in vitro*. Second, we showed that inhibition of mTOR signalling by everolimus induced G_0_/G_1_ arrest and apoptosis, suggesting that everolimus might inhibit anti-apoptotic or survival signalling in OSCC cell lines. Third, we found that treatment with everolimus significantly inhibited tumour growth *in vivo*. Taken together, these results indicate that everolimus as a single agent could have significant anti-tumour efficacy against OSCC cells.

The effects of everolimus were more prominent in TE11 cells (p-mTOR-low) than in TE4 cells (p-mTOR-high). The malignant characteristics of OSCC cells are likely acquired not only through the mTOR signalling pathway but also through a wide variety of other signalling pathways. Although the activation level of the mTOR pathway was lower in TE11 cells than in TE4 cells, TE11 cells might depend more heavily on the mTOR pathway for their malignant behaviour. On the other hand, although TE4 cells showed a high level of mTOR activation, they might rely more on the other signalling pathways than on the mTOR pathway. In this study, the mTOR pathway was activated in all five cell lines assessed. If we could obtain an OSCC cell line without mTOR activation, those cells might well be resistant to everolimus. Future studies are necessary to confirm our findings as well as to elucidate the biological mechanisms by which the mTOR activation level affects the therapeutic efficacy of everolimus.

Interestingly, we also found an additive effect of everolimus and cisplatin on OSCC cells in an *in vivo* model. A similar effect has been reported for other types of carcinomas. Beuvink *et al*, (2005) reported that everolimus could sensitise cells to cisplatin by inhibiting induction of p21 expression by p53. Ma *et al*, (2010) showed that everolimus exerts an additive-to-synergetic effect on cisplatin-induced growth inhibition in nasopharyngeal carcinoma. Unfortunately, our current experiment could show only an additive effect rather than a synergetic effect. We expect that additional experiments in the future might be able to show a synergetic effect. However, we were at least able to recognise that combining everolimus and cisplatin might be a useful therapeutic strategy. As cisplatin is one of the most important chemotherapeutic drugs for OSCC treatment, our finding may have significant clinical implications.

In conclusion, most OSCC tumours showed mTOR activation, suggesting that mTOR could be a promising target for anti-cancer therapy against OSCC. Everolimus had a therapeutic effect on OSCC cells both *in vitro* and *in vivo*, and combination therapy with everolimus and cisplatin showed an additive effect. Although further experimental studies are necessary to confirm our findings, the current study certainly provides further rationale for future clinical trials of everolimus (in combination with cisplatin) in OSCC patients.

## Figures and Tables

**Table 1:** Effect of everolimus on tumour development of TE4 cell lines

**Treatment**	**Number of mice**	**Mean tumour volume (mm^3^)**	**% Effect of tumour reduction**
Placebo	6	1314±134	100
Everolimus	5	311±87^*^^,†^	23
Cisplatin	5	542±161^*^	41
Everolimus+cisplatin	6	159±21^*^^,†,‡^	12

^*^Significantly different from the placebo group (*P*<0.05).

^†^Significantly different from the cisplatin group (*P*<0.05).

^‡^Significantly different from the everolimus group (*P*<0.05).
