# Supplementary figures and images for: Aberrant activation of the mTOR pathway and anti-tumour effect of everolimus on oesophageal squamous cell carcinoma
Source: Br J Cancer. 2012 Feb 14;106(5):876–82. doi: 10.1038/bjc.2012.36 (PMC3305959; doi:10.1038/bjc.2012.36)

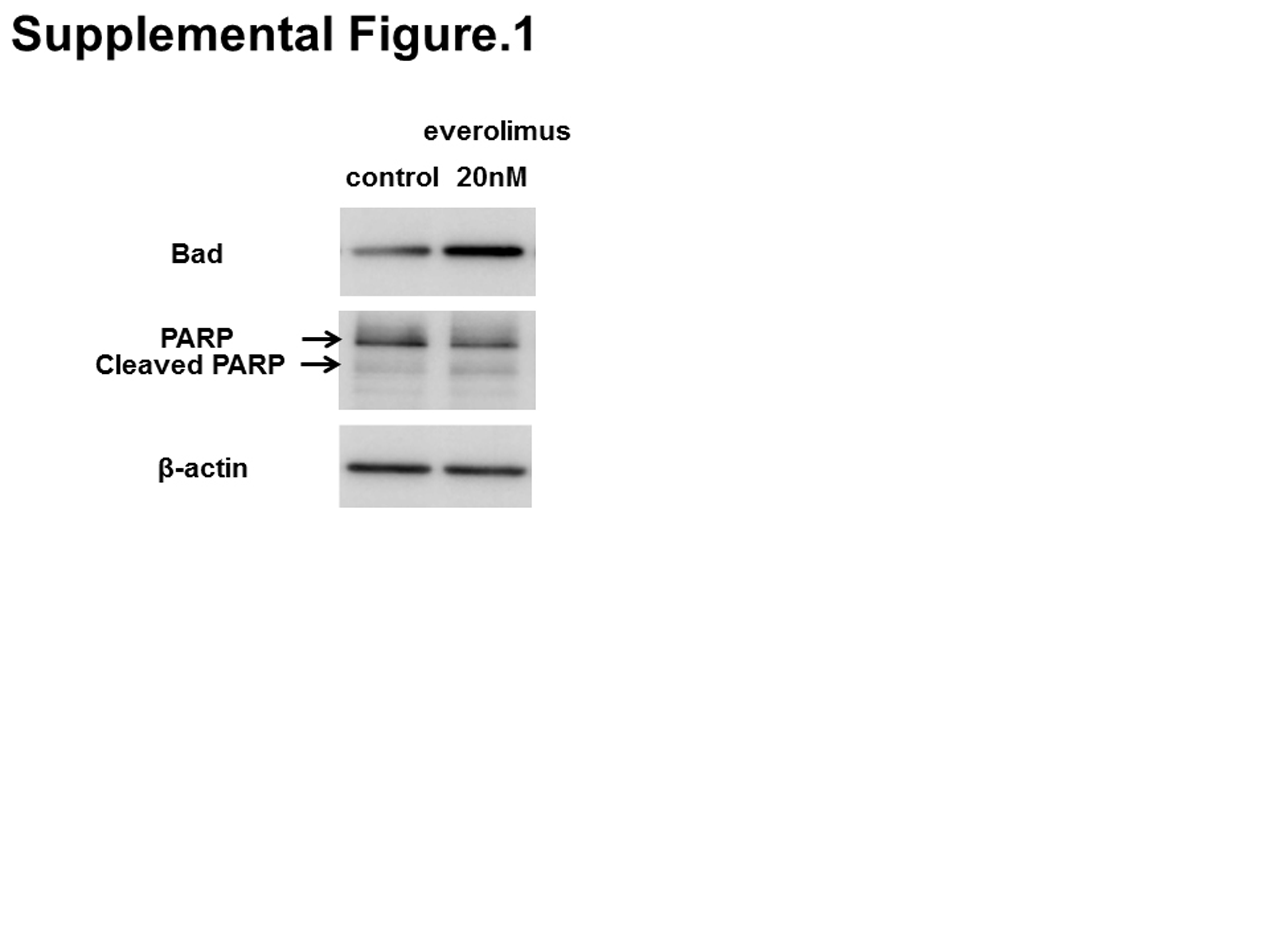

Supplement: Supplementary Figure 1 [file bjc201236x1.tif]

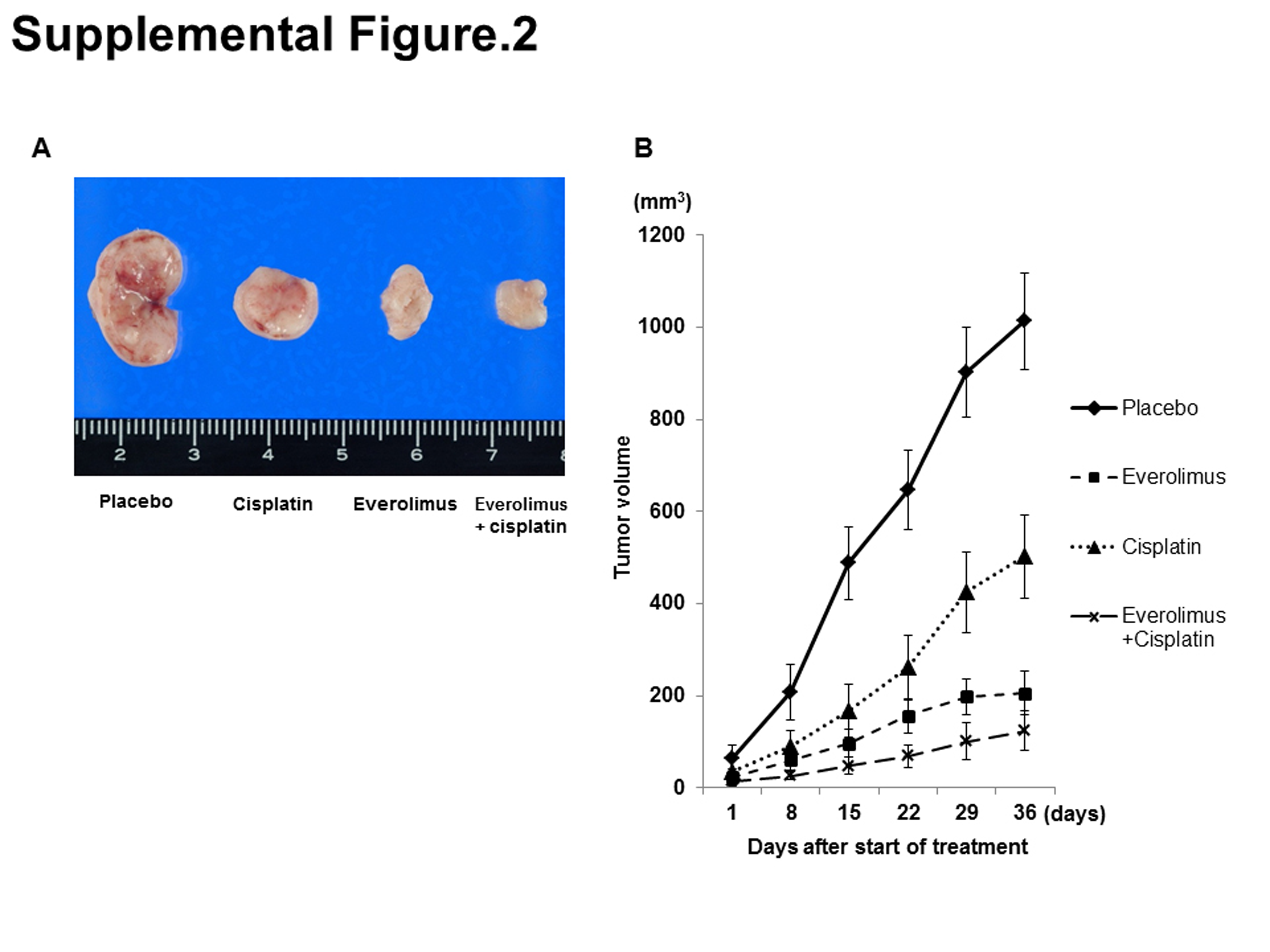

Supplement: Supplementary Figure 2 [file bjc201236x2.tif]

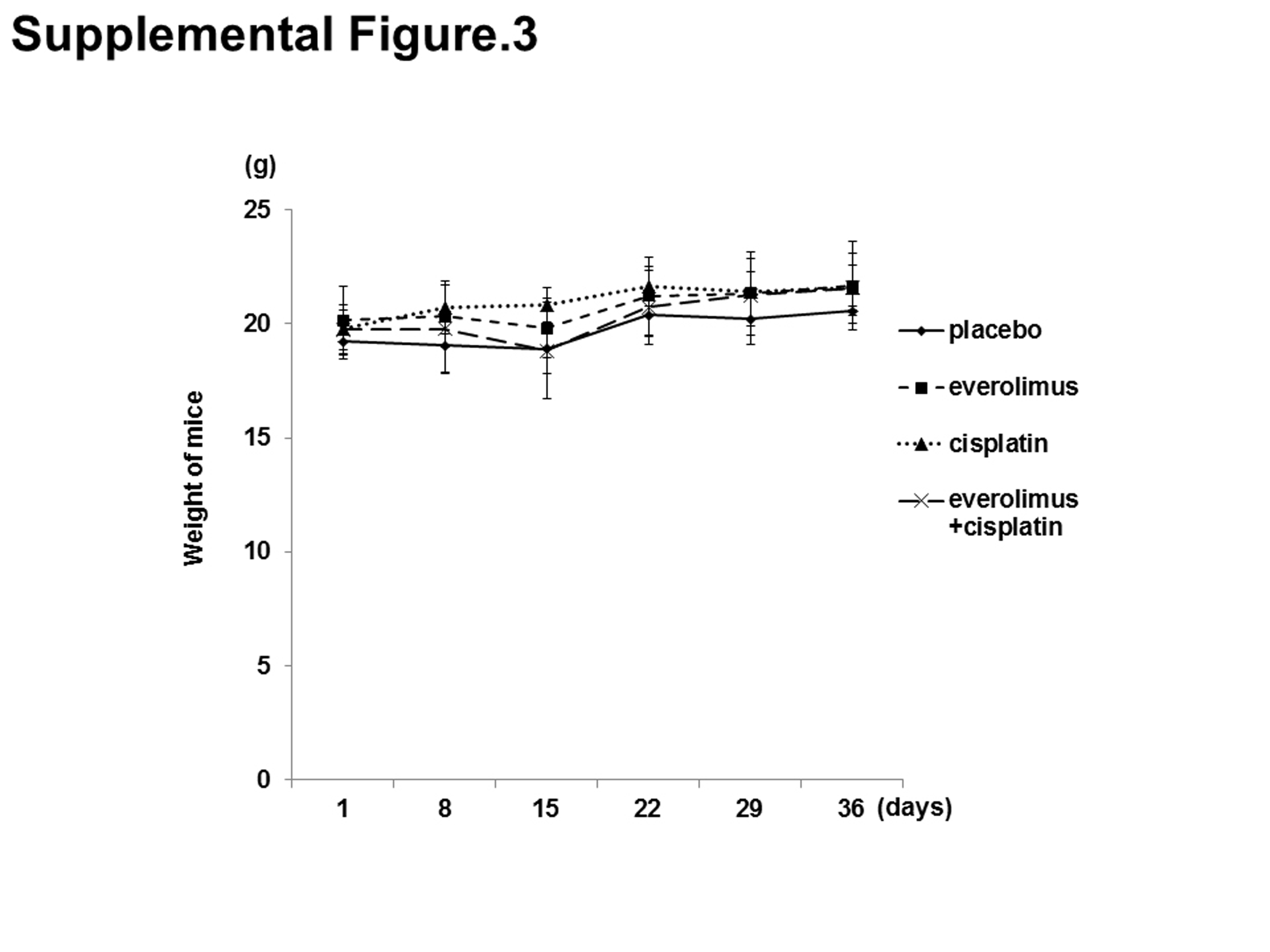

Supplement: Supplementary Figure 3 [file bjc201236x3.tif]

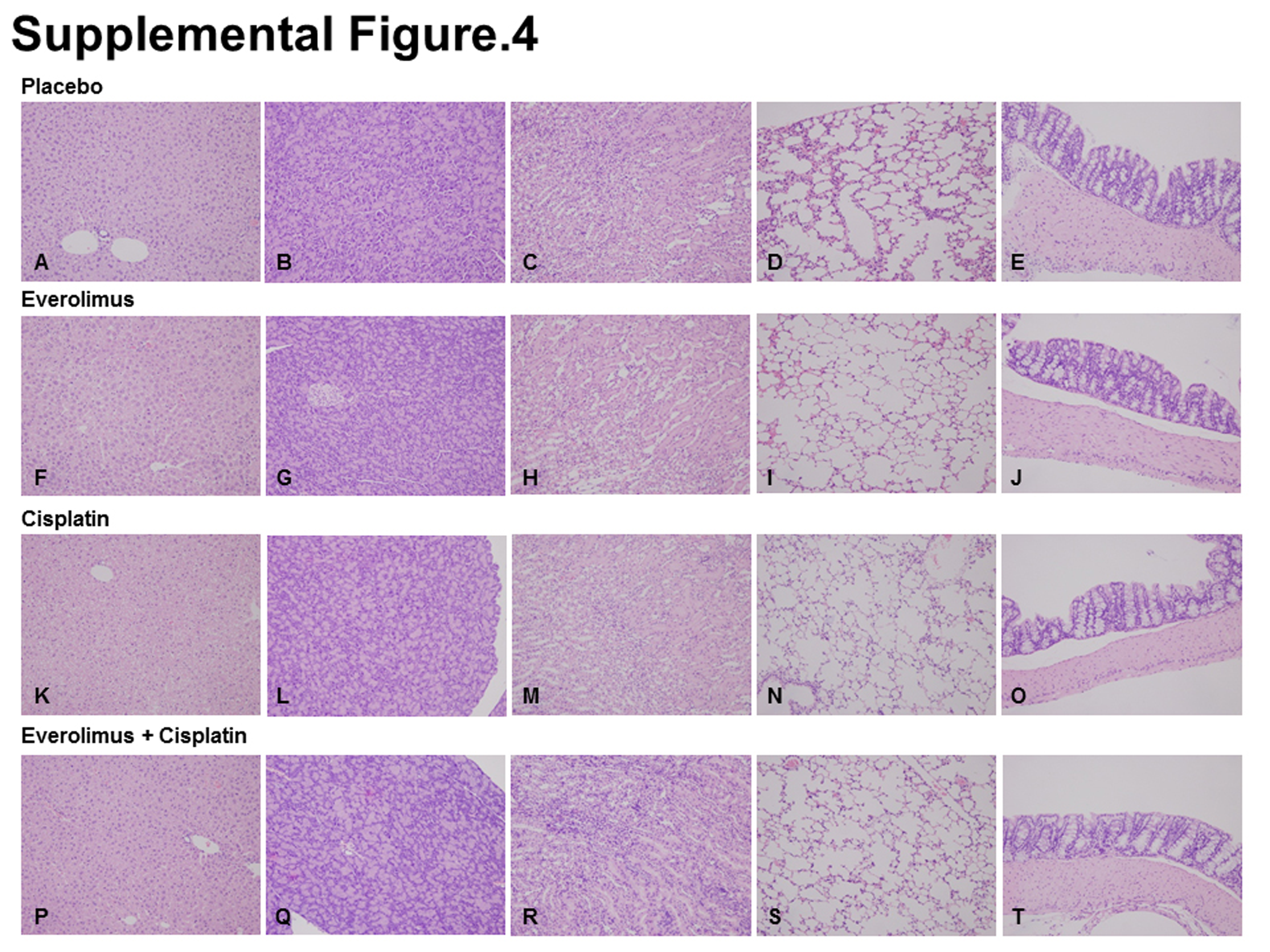

Supplement: Supplementary Figure 4 [file bjc201236x4.tif]
