# Supplementary material for: Aberrant activation of the mTOR pathway and anti-tumour effect of everolimus on oesophageal squamous cell carcinoma
Source: Br J Cancer. 2012 Feb 14;106(5):876–82. doi: 10.1038/bjc.2012.36 (PMC3305959; doi:10.1038/bjc.2012.36)
Supplement: Supplementary Figure Legends [file bjc201236x5.doc]

**Supplemental**

**Supplemental Figure 1**

Western blot analysis for Bad and PERP in TE4 cells treated with everolimus (20nM).

**Supplemental Figure 2:** In vivo assay for confirming the anti-cancer activity of everolimus utilizing a mouse xenograft model established with TE11 cells.

A: Tumour volume in the 4 treatment groups (placebo, everolimus, cisplatin, and everolimus plus cisplatin) after the 5-week course of treatment.

B: Growth of tumour volume in the 4 treatment groups.

**Supplemental Figure 3**

The weight changes of the mice in the 4 treatment groups (placebo, everolimus, cisplatin, and everolimus plus cisplatin) during the 5-week course of treatment. The mean day-36 weights of mice treated with placebo, everolimus, cisplatin, and everolimus plus cisplatin were 19.8 ± 0.83 mm3, 21.9 ± 1.78 mm3, 21.6 ± 1.35 mm3, and 21.8 ± 0.93 mm3, respectively. There was no significant difference among the 4 groups.

**Supplemental Figure 4**

Histological evaluation of organ injury (**A, F, K, P**: liver, **B, G, L, Q**: pancreas, **C, H, M, R**: kidney, **D, I, N, S**: lung, and **E, J, O, T**: intestine) in the mice in the 4 treatment groups (**A–E**: placebo, **F–J**: everolimus, **K–O**: cisplatin, **P–T**: everolimus plus cisplatin).
